# Analysis of clinical characteristics of elderly patients with blood culture-positive bacterial liver abscess

**DOI:** 10.3389/fmed.2025.1555056

**Published:** 2025-03-20

**Authors:** Hui-Fang Zhang, Jia-Wen Chen, Shan-Shan Li, Shi-Wen Wu, Shu Li, Chen-Yi Liu, Chao Cai, Ming-Qin Lu

**Affiliations:** Department of Infectious Diseases, The First Affiliated Hospital of Wenzhou Medical University, Wenzhou, Zhejiang, China

**Keywords:** elderly, blood culture-positive, bacterial liver abscess, clinical characteristics, *Klebsiella pneumoniae*

## Abstract

**Objective:**

To analyze the clinical features of elderly patients with blood culture-positive bacterial liver abscess (BLA) and improve diagnostic and treatment strategies.

**Methods:**

Elderly BLA patients admitted to our hospital from December 2018 to December 2023 were included in the study. Diagnostic tests included routine blood analysis, biochemistry, C-reactive protein (CRP), procalcitonin (PCT), imaging, and cultures of blood or pus. Treatments involved anti-infective therapy, ultrasound-guided abscess drainage, and supportive care.

**Results:**

(1) Elderly patients with blood culture-positive BLA had higher rates of prolonged hospital stays (≥2 weeks), ICU admission, biliary system diseases, hepatitis B infection, maximum body temperature ≥ 39°C, and qSOFA scores ≥2 compared to controls (*p* < 0.05)0. (2) Laboratory findings showed higher levels of total bilirubin (≥34.2 μmol/L), ALT (≥50 U/L), serum creatinine (≥80 μmol/L), PCT (≥5 ng/mL), and lower platelet counts (≤100 × 10^9^/L) in the research group (*p* < 0.05). ESBL-positive cases and liver abscesses ≤5 cm were more common in the research group (*p* < 0.05). (3) Complications such as pleural effusion, ascites, pulmonary infections, and extrahepatic abscesses were significantly more frequent in the blood culture-positive group (*p* < 0.05). (4) Microbiological analysis indicated that *Klebsiella pneumoniae* was the leading pathogen (87.93%), followed by *Escherichia coli*. For ESBL-positive infections, *E. coli* was dominant (75.76%), especially in patients with biliary diseases (75.56%). (5) Logistic regression identified prolonged hospital stay, hepatitis B infection, biliary system diseases, temperature ≥ 39°C, PCT ≥5, and abscess size ≤5 cm as independent risk factors for blood culture-positive BLA. (6) The combined diagnostic indicator showed good predictive ability (AUC = 0.840, sensitivity 76.6%, specificity 72.2%).

**Conclusion:**

Elderly patients with biliary diseases, hepatitis B, high PCT levels (≥5 ng/mL), small abscesses (≤5 cm), and fever (≥39°C) are at higher risk for blood culture-positive BLA. *Klebsiella pneumoniae* remains the predominant pathogen (87.93%), highlighting the need for prompt empirical antibiotic therapy. The combined diagnostic model offers reliable predictive value for this condition. We developed a predictive model aimed at assisting clinicians in identifying high-risk patients prone to bloodstream infections secondary to BLA. This model provides valuable guidance for clinicians in formulating more rational and individualized treatment strategies.

## 1 Introduction

Bacterial liver abscess (BLA) is a localized necrotizing disease characterized by liquefactive necrosis of liver tissue resulting from the invasion of pathogenic bacteria. The incidence of BLA has been increasing annually. BLA presents with a range of non-specific symptoms, such as fever, upper right abdominal pain, nausea, and vomiting. The clinical manifestations of liver abscesses have also changed over the years because of the overuse of antibiotics, with an increased incidence of resistance to bacteria as well as a higher prevalence of patients with chronic or malignant diseases, likely because of aging populations around the world. (1, 2). When a liver abscess is complicated by severe conditions such as bloodstream infections, sepsis, septic shock, and multiple organ failure, failure to receive timely diagnosis and treatment may pose a significant threat to the patient’s life (3, 4). Bloodstream infection, as one of the common complications in patients with bacterial liver abscess, has a critical impact on the progression of the disease and the prospects for recovery (5). This study selected 290 elderly patients with BLA admitted to our hospital from December 2018 to December 2023, including 145 patients with positive blood cultures and 145 patients with negative blood cultures. The aim was to discuss the clinical characteristics of elderly patients with bacteriological liver abscess and the risk factors associated with the occurrence of the disease, in order to provide a scientific basis for clinical prevention and treatment. Our study has also developed a predictive model to assess the likelihood of secondary bloodstream infections in elderly patients with liver abscess.

## 2 Materials and methods

### 2.1 General information

Among the patients with BLA at our hospital, we screened 343 elderly patients diagnosed and treated from December 2018 to December 2023. Following a rigorous review of data completeness, 11 patients with incomplete records were excluded. Propensity score matching was performed based on age and sex, resulting in the inclusion of 290 patients in the final analysis, comprising 182 males and 108 females. Based on the results of blood cultures and drainage fluid bacterial cultures (where the same bacterial species was identified in both the blood and drainage fluid cultures of the same patient), patients were categorized into a blood culture-positive group and a blood culture-negative group. All patients received standard antibacterial treatment, along with percutaneous abscess drainage, as well as other necessary symptomatic supportive care. The patients included in this study were required to meet the following criteria: (1) patients who presented to our hospital for their initial consultation after disease onset and were aged >60 years; (2) presence of typical symptoms such as fever, and a diagnosis of liver abscess confirmed through imaging examinations; (3) positive results in blood cultures and/or abscess puncture fluid cultures. The exclusion criteria included: (1) patients with positive bacterial culture results indicating infections caused by special pathogens such as fungi or *Mycobacterium tuberculosis*; (2) the clinical and diagnostic records of the patient are incomplete. This study has received formal approval from the Ethics Committee of the First Affiliated Hospital of Wenzhou Medical University (Ethics Approval Number: KY2024-R254).

### 2.2 Methods

We systematically collected and organized a series of key information regarding patients through the hospital’s electronic medical record system. This information encompasses the patients’ demographic data. Such information includes general demographics, hematological test data, and results of other specific assays. These assays include C-reactive protein (CRP) and procalcitonin (PCT) tests. Additionally, bacterial culture results, radiological findings, patients’ symptoms and signs, comorbidities, and details of underlying diseases are also included.

### 2.3 Statistical analysis

This study utilized SPSS 27 statistical software for comprehensive data analysis and processing. For continuous data that were normally distributed, we presented the results as means and standard deviations (*x* ± *s*) and applied *t*-tests to assess differences. For data that did not conform to a normal distribution, we represented the results using medians and interquartile ranges [*M* (Q25, Q75)] and employed the Mann–Whitney *U* test to evaluate differences. Categorical data were expressed as percentages (%) and analyzed using the *χ*^2^ (chi-square) test to determine statistical significance. The core of the study lies in comparing the clinical characteristics of elderly patients with BLA who tested positive for blood cultures versus those who tested negative. Based on this comparison, we conducted a thorough analysis of the factors associated with positive blood cultures in elderly patients with BLA. In addition, we employed a logistic regression model to further identify the independent risk factors associated with positive blood cultures in elderly patients with BLA. A *p*-value of less than 0.05 was considered to indicate statistical significance. To provide a more intuitive assessment of the predictive efficacy of each indicator, we also constructed receiver operating characteristic (ROC) curves, which served as a reference for a comprehensive evaluation of the predictive value of the various indicators. Combined Index = 1.301 × (Liver Abscess ≤5 cm) + (−0.947) × (Coexisting Biliary Tract Disease) + (−1.514) × (Coexisting Hepatitis B Virus Infection) + 1.013 × (Maximum Temperature ≥ 39°C upon Admission) + 1.999 × (PCT ≥ 5 ng/mL) + (−0.364).

## 3 Results

### 3.1 Comparison of general characteristics and clinical conditions between the two groups

The two groups of patients were comparable in terms of age, sex, and composition, as well as the incidence of diabetes, hypertension, and malignant tumors, with no statistically significant differences observed (*p* > 0.05). However, the bloodstream-positive group exhibited a higher prevalence of ICU admission, coexisting biliary tract diseases, coexisting hepatitis B virus infection, length of hospital stay ≥2 weeks, maximum temperature ≥ 39°C upon admission, and qSOFA score ≥ 2 compared to the control group, with statistically significant differences noted (*p* < 0.05) (Tables 1, 2).

**Table 1:** The general characteristics and ICU admission status of elderly patients with BLA in both groups.

Item		Blood culture negative *n* = 145	Blood culture positive *n* = 145	*χ*^2^/*Z*	*P*
Sex	Male	91(54.5)	102(65.4)	3.98	0.046
	Female	76(45.5)	54(34.6)		
Age		69(65,76.25)	69(65,74)	−0.150	0.88
Diabetes	Yes	89(53.3)	83(53.2)	0	0.987
	No	78(46.7)	73(46.8)		
Hypertension	Yes	69(41.3)	69(44.2)	0.280	0.597
	No	98(58.7)	87(55.8)		
ICU Admission	Yes	17(10.2)	59(37.8)	34.648	<0.001
	No	150(89.8)	97(62.2)		
Biliary System Disease	Yes	56(33.5)	78(50)	9.01	0.003
	No	111(66.5)	78(50)		
Malignant Tumor	Yes	21(12.6)	21(13.5)	0.056	0.813
	No	146(87.4)	135(86.5)		
Hepatitis B Virus Infection	Yes	30(18.0)	67(42.9)	23.961	<0.001
	No	137(82.0)	89(57.1)		
qSOFA ≥2		9(5.4)	27(17.3)	11.569	<0.001
Highest temperature on admission ≥39°C		75(44.9)	99(63.5)	11.17	<0.001
Mortality rate (%)		2(1.2)	6(3.8)	2.342	0.126

**Table 2:** The general conditions and ICU admission status of elderly BLA patients in the two groups after propensity matching.

Item		Blood culture negative *n* = 145	Blood culture positive *n* = 145	*χ*^2^/*Ζ*	*P*
Gender	Male	91(62.8)	91(62.8)	0	1
	Female	54(37.2)	54(37.2)		
Age		69(64,74.5)	69(65,74)	−0.475	0.635
Diabetes	Yes	82(56.6)	78(53.8)	0.223	0.673
	No	63(43.4)	67(46.2)		
Hypertension	Yes	60(41.4)	64(44.1)	0.225	0.635
	No	85(58.6)	81(55.9)		
ICU admission	Yes	13(9)	57(39.3)	36.457	<0.001
	No	132(91)	88(60.7)		
Biliary system disease	Yes	45(31)	74(51)	11.985	<0.001
	No	100(69)	71(49)		
Malignant Tumor	Yes	18(12.4)	19(13.1)	0.031	0.86
	No	127(87.6)	126(86.9)		
Hepatitis B virus infection	Yes	22(15.2)	63(43.4)	27.986	<0.001
	No	123(84.8)	82(56.6)		
qSOFA ≥2		6(4.1)	27(18.6)	15.081	<0.001
Highest temperature on Admission ≥39°C		65(44.8)	93(64.1)	10.901	<0.001
Mortality rate (%)		2(1.4)	6(4.1)	2.057	0.152

### 3.2 Significant differences in laboratory indicators between two groups

The bloodstream-positive group exhibited significantly higher counts in total bilirubin ≥34.2, ALT ≥50, serum creatinine ≥80, PCT ≥ 5, platelet count ≤100, and the number of ESBL-positive cases compared to the control group, with statistically significant differences observed (*p* < 0.05) (Table 3).

**Table 3:** The laboratory test parameters of elderly patients with BLA in both groups.

Item	Blood culture negative *n* = 145	Blood culture positive *n* = 145	*χ* ^2^	*P*
Hospitalization duration ≥2 weeks	70(48.3)	99(68.3)	11.927	<0.001
White blood cell count ≥10 × 10^9^/L	108(74.5)	103(71.0)	0.435	0.51
Fasting blood glucose >7 mmol/L	128(88.3)	121(83.4)	1.392	0.238
Total bilirubin ≥34.2 μmol/L	19(13.1)	43(29.7)	11.817	<0.001
Neutrophil percentage ≥ 0.7	138(95.2)	139(95.9)	0.081	0.777
ALT ≥50 U/L	76(52.4)	98(67.6)	6.954	0.008
Creatinine ≥80 μmol/L	50(34.5)	87(60.0)	18.941	<0.001
Sodium ≤135 mmol/L	82(56.6)	81(55.9)	0.014	0.906
Platelet count ≤100 × 10^9^/L	23(15.9)	57(39.3)	19.955	<0.001
Albumin ≤35 g/L	15(10.3)	17(11.7)	0.141	0.708
Hemoglobin ≤110 g/L	58(40.0)	43(29.7)	3.418	0.064
CRP ≥ 90 mg/L	121(83.4)	122(84.1)	0.025	0.873
PCT ≥ 5 ng/mL	50(34.5)	108(74.5)	46.776	<0.001
ESBL positive	9(6.2)	24(16.6)	7.694	0.006

### 3.3 Comparison of liver abscess size and related characteristics between the two groups

The proportion of liver abscesses measuring between 5 and 10 cm was significantly lower in the bloodstream-positive group compared to the control group, while the proportion of liver abscesses ≤5 cm was significantly higher in the bloodstream-positive group than in the control group, with statistically significant differences noted (*p* < 0.05). Conversely, there were no statistically significant differences observed between the two groups regarding liver abscesses ≥10 cm, the number of liver abscesses, their locations, and the presence of septations within the abscesses (*p* > 0.05) (Table 4).

**Table 4:** The characteristics of size and location of BLA in elderly patients in both groups.

Item	Blood culture negative *n* = 145	Blood culture positive *n* = 145	*χ* ^2^	*P*
Abscess size ≤5 cm	24 (16.5)	58 (40.0)	19.665	<0.001
5 cm < Abscess size <10 cm	101(69.7)	74(51.0)	10.505	0.001
Abscess size ≥10 cm	20(13.8)	13(9.0)	1.676	0.196
Number of abscesses ≥2	28(19.3)	37(25.5)	1.606	0.205
Presence of septation	116(80)	112(77.2)	0.328	0.567
Presence of septation			1.695	0.429
Right lobe	90(62.1)	93(64.1)		
Left lobe	53(36.6)	47(32.4)		
Junction of both lobes	2(1.4)	5(3.5)		

### 3.4 Comparison of complication occurrence between the two groups

The incidence of pleural effusion, ascites, pulmonary infection, and extrahepatic abscesses was significantly higher in the bloodstream-positive group compared to the control group, with statistically significant differences observed (*p* < 0.05) (Table 5).

**Table 5:** The incidence of complications in two groups of elderly patients with BLA.

Item	Blood culture negative *n* = 145	Blood culture positive *n* = 145	*χ* ^2^	*P*
Pleural effusion (yes)	28 (19.3)	57 (39.3)	13.997	<0.001
Abdominal effusion (yes)	15 (10.3)	27 (18.6)	4.009	0.045
Pulmonary infection (yes)	16 (11.0)	33 (22.8)	7.097	0.008
Endophthalmitis (yes)	3 (2.1)	1 (0.7)	1.014	0.314
Extracapsular abscess (yes)	6 (4.1)	20 (13.8)	8.281	0.004

### 3.5 Clinical characteristics and bacteriological analysis of PLA patients with ESBL-positive infections

(1) Bacteriological classification revealed that both groups were predominantly comprised of *Klebsiella pneumoniae* (87.93%), followed by *Escherichia coli*. (2) Among the cases that tested positive for extended-spectrum beta-lactamases (ESBL), *E. coli* was the most common pathogen (75.76%). Furthermore, patients with ESBL-positive infections were more frequently observed to have underlying biliary tract diseases (81.82%) (3) qSOFA score ≥ 2, presence of pleural effusion, ascites, total bilirubin ≥34.2 μmol/L, platelet count ≤100 × 10^9^/L, and positive blood culture rates were significantly higher in BLA patients with ESBL-positive infections compared to those with ESBL-negative infections, with statistically significant differences (*p* < 0.05) (Tables 6–8).

**Table 6:** Distribution of pathogenic microorganisms in elderly patients with BLA.

Bacterial classification		Positive blood culture group (*n* = 145)		Total cases	Negative blood culture group (n = 145)	Total cases	Proportion (100%)
		Positive blood culture only	Positive in blood and drainage fluid				
*Klebsiella pneumoniae*		39	72	121	134	255	87.93%
*Escherichia coli*		12	7	19	11	30	10.35%
Mixed infection (*Escherichia coli* + Enterococcus)		2	1	3	0	3	1.72%
Mixed infection (*Escherichia coli* + Streptococcus)		2	0	2	0	2	
ESBL Positive	*Klebsiella pneumoniae*	4	2	6	2	33	24.24%
	*Escherichia coli*	10	8	18	7		75.76%

**Table 7:** The distribution of bacteria and major underlying comorbidities in elderly patients with ESBL-positive BLA.

Bacterial classification		Diabetes		Biliary disease		ICU admission		Malignant tumor	
ESBL-positive (33)	*Klebsiella pneumoniae* (8)	4	39.39%	5	81.82%	3	39.39%	1	39.39%
	*Escherichia coli* (25)	9		20		10		12	

**Table 8:** Comparison of underlying diseases, complications, and laboratory indicators between ESBL-positive and ESBL-negative elderly BLA patients.

Item	ESBL-negative patients (*n* = 257)	ESBL-positive patients (*n* = 33)	*χ* ^2^	*P*-value
Diabetes (yes)	147(57.2)	13(39.4)	3.748	0.053
Biliary tract diseases (Yes)	92(35.8)	27(81.8)	25.598	<0.001
Hypertension (yes)	111(43.2)	13(39.4)	0.172	0.678
Malignancy (yes)	24(9.3)	18(39.4)	23.734	<0.001
qSOFA ≥2	25(9.7)	8(24.2)	6.110	0.013
Hospital stay ≥2 weeks	146(56.8)	23(69.7)	1.998	0.158
WBC ≥ 10 × 10^9^/L	190(73.9)	21(63.6)	1.563	0.211
Total bilirubin ≥34.2 μmol/L	48(18.7)	14(42.4)	9.812	0.002
ALT ≥50 U/L	157(61.1)	17(51.5)	1.117	0.291
Creatinine ≥80 mmol/L	121(47.1)	16(48.5)	0.023	0.879
Platelet count ≤100 × 10^9^/L	65(25.3)	15(45.5)	5.592	0.015
Highest admission temperature ≥ 39°C	143(55.6)	15(45.5)	1.224	0.269
Albumin ≤35 g/L	29(11.3)	3(9.1)	0.143	0.705
CRP ≥ 90 mg/L	219(85.2)	24(72.7)	3.358	0.067
PCT ≥ 5 ng/mL	136(52.9)	22(66.7)	2.229	0.135
Positive blood culture rate	121(42.1)	24(72.7)	7.694	0.006
Pleural effusion (yes)	68(26.5)	17(51.5)	8.881	0.003
Ascites (yes)	31(12.1)	11(33.3)	10.684	0.001
Extrahepatic abscess (yes)	24(9.3)	2(6.1)	0.385	0.535
Pulmonary infection (yes)	42(16.3)	7(21.2)	0.494	0.482
Endophthalmitis (yes)	3(1.2)	1(3.0)	0.746	0.388
Mortality rate	6(2.3)	2(6.1)	1.514	0.219

### 3.6 Analysis of risk factors for positive blood culture bacterial liver abscess in elderly patients

With blood culture results as the dependent variable (positive = 1, negative = 0), the following factors were included in the analysis: admission to the intensive care unit (yes), length of hospital stay ≥2 weeks, biliary tract disease (present), hepatitis B virus infection (present), pleural effusion (present), ascites (present), pulmonary infection (present), extrahepatic abscess (present), ALT ≥50, serum creatinine ≥80, PCT ≥5, platelet count ≤100, maximum temperature after admission ≥39°C, qSOFA score ≥ 2, and liver abscess size ≤5 cm. Logistic regression analysis was employed to identify the factors influencing positive bacterial blood cultures in elderly patients with bacterial liver abscesses. The results indicated that a hospital stay of ≥2 weeks, coexisting hepatitis B virus infection, presence of biliary tract disease, maximum temperature after admission ≥39°C, PCT ≥5, and abscess size ≤5 cm were identified as independent risk factors influencing the occurrence of positive bacterial blood cultures in elderly patients with bacterial liver abscesses (Tables 9, 10).

**Table 9:** Univariate logistic analysis of factors influencing the occurrence of positive blood cultures in elderly patients with BLA.

Item	*B*	*P*-value	OR value	95% confidence interval	
				Lower limit	Upper limit
Length of hospital stay ≥14 Days	0.652	0.045	1.92	1.015	3.632
ICU admission (yes)	0.711	0.138	2.035	0.796	5.201
Biliary system disease (Yes)	1.07	0.002	2.915	1.493	5.693
Hepatitis B virus infection (Yes)	1.428	<0.001	4.17	2.043	8.512
Pleural effusion (yes)	0.787	0.066	2.197	0.949	5.087
Abdominal effusion (yes)	−0.394	0.463	0.674	0.235	1.932
Pulmonary infection (yes)	−0.459	0.373	0.632	0.23	1.734
Extrahepatic abscess (Yes)	0.653	0.353	1.922	0.485	7.618
Total bilirubin ≥34.2 μmol/L	0.727	0.105	2.069	0.859	4.985
ALT ≥50 U/L	0.465	0.182	1.592	0.804	3.152
Creatinine ≥80 μmol/L	0.603	0.068	1.827	0.956	3.493
PCT ≥ 5 ng/mL	1.571	<0.001	4.812	2.375	9.751
Platelet count ≤100 × 10^9/L	0.025	0.949	1.025	0.474	2.218
Abscess size ≤5 cm	1.361	<0.001	3.898	1.884	8.067
qSOFA ≥2	0.549	0.424	1.731	0.452	6.635
Maximum temperature ≥ 39°C (after admission)	1.032	0.002	2.806	1.469	5.36

**Table 10:** The multivariable logistic analysis of factors influencing the incidence of positive blood cultures in elderly patients with BLA.

	*B*	P-value	OR value	95% confidence interval	
				Lower limit	Upper limit
Length of hospital stay ≥14 days	0.365	0.038	1.440	1.021	2.073
Biliary system disease (yes)	0.396	0.026	1.486	1.052	2.189
Hepatitis B virus infection (Yes)	0.524	0.005	1.688	1.178	2.499
PCT ≥ 5 ng/mL	0.721	<0.001	2.057	1.451	3.069
Abscess size ≤5 cm	0.512	0.005	1.669	1.178	2.488
Maximum body temperature after admission ≥39°C	0.553	0.002	1.738	1.229	2.603

### 3.7 Diagnostic efficacy of PCT and combined indicators for blood culture positive bacterial liver abscess

**Figure 1:**
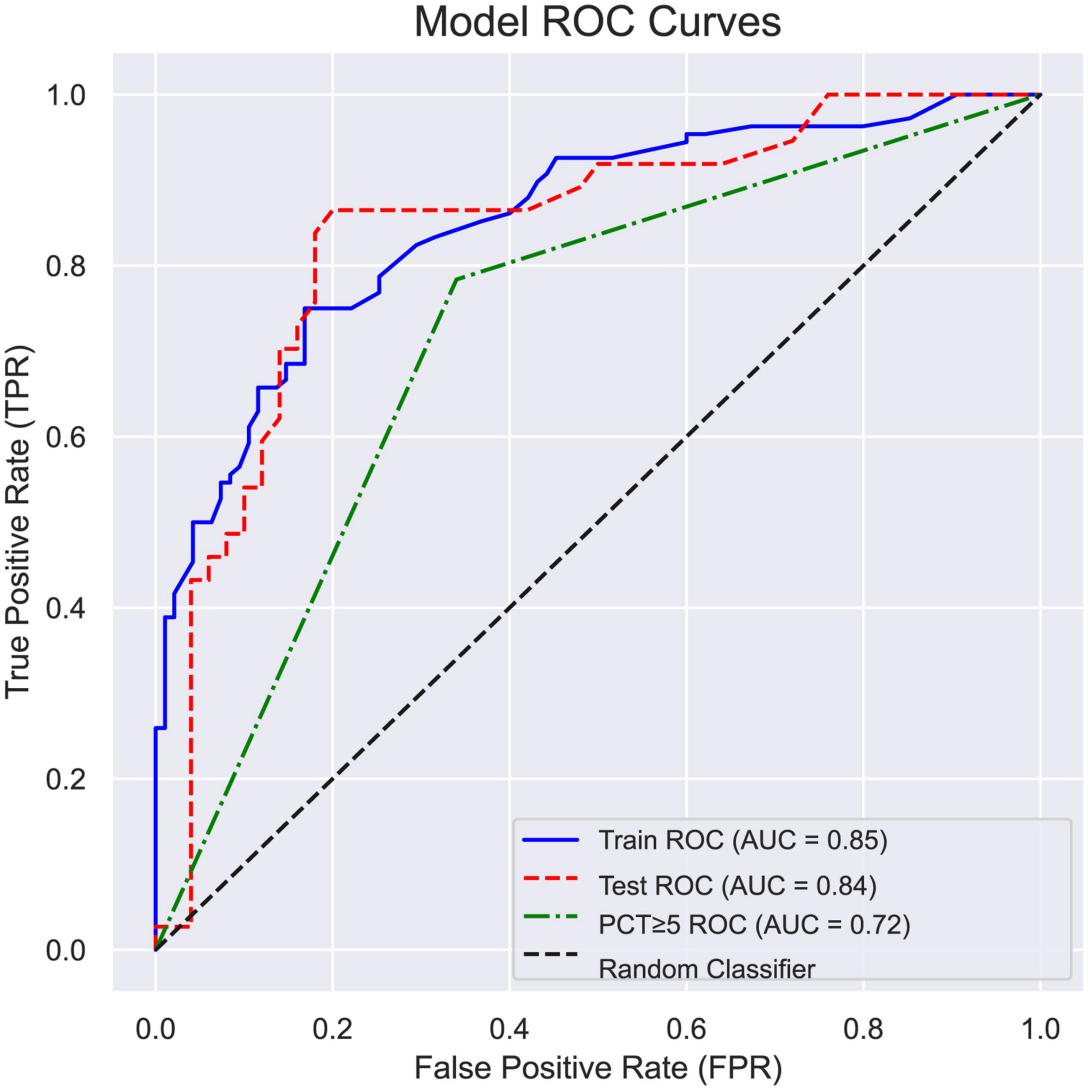
Receiver operating characteristic (ROC) curve of factors affecting the occurrence of positive blood cultures in patients with bacterial liver abscess.

The area under the curve (AUC) for PCT ≥5 was 0.700 with a 95% confidence interval (CI) of 0.647–0.753; the sensitivity was 0.745 and the specificity was 0.655. In contrast, the AUC for the combined indicators was 0.840 with a 95% CI of 0.780–0.873; the sensitivity was 0.766 and the specificity was 0.722 (Table 11 and Figure 1).

**Table 11:** Area under the curve (AUC) for factors influencing the incidence of positive blood cultures in elderly patients with BLA.

Test result variable	AUC	*P*	95% confidence interval		Sensitivity	Specificity
			Lower limit	Upper limit		
PCT ≥ 5	0.722	<0.001	0.611	0.833	0.784	0.660
Combined indicators	0.841	<0.001	0.752	0.930	0.865	0.800

## 4 Discussion

Bacterial liver abscess is an acute infectious condition caused by the invasion of pyogenic bacteria into the liver (6). This disease is particularly prevalent among diabetic patients and the elderly population, and it is prone to complications such as bloodstream infections, sepsis, and septic shock. Delayed diagnosis or insufficient timely treatment may ultimately result in severe outcomes, including patient mortality. With the acceleration of population aging, the prevalence of diabetes and cancer patients has been steadily increasing, coupled with the widespread use of immunosuppressive agents, resulting in a rising incidence of BLA (7). Elderly individuals often suffer from multiple underlying health conditions, experience a decline in physiological function, and exhibit weakened immune responses. Furthermore, the diverse clinical manifestations of BLA, combined with a lack of distinct specificity, pose significant challenges for the early diagnosis and treatment of this condition (8). In recent years, the incidence of BLA has increased compared to previous years, with a notably high recurrence rate, particularly among elderly patients with multiple underlying health conditions (1, 6). Previous studies have identified that diabetic patients, individuals with biliary diseases, and those with hypertension are the primary populations affected by BLA (9–11), which is consistent with the findings of this study. This study further indicates that biliary disease is an independent risk factor for bloodstream infections in elderly patients with BLA. According to the literature, biliary diseases are not only a primary pathogenic cause of BLA but also a significant predictor of BLA recurrence, particularly in patients with a history of cholangitis (12). Moreover, this study also found that infection with hepatitis B virus (HBV) is an independent risk factor for bloodstream-positive BLA in the elderly. This association may be related to the tendency for HBV infection to progress to chronic hepatitis, liver cirrhosis, and even hepatocellular carcinoma. These pathological conditions may lead to the development of portal hypertension, portal vein thrombosis, or tumor thrombus, all of which are closely linked to the pathways of BLA formation, primarily through the biliary tract, hepatic artery, and portal vein.

This study emphasizes that for elderly patients presenting with high fever and a quick Sequential Organ Failure Assessment (qSOFA) score of 2 or higher, the presence of a liver abscess on imaging should raise a high suspicion for the possibility of bloodstream infection. It is imperative to closely monitor changes in the patient’s vital signs while actively implementing appropriate antimicrobial therapy and supportive symptomatic treatment measures. The study also found that the levels of bilirubin, alanine aminotransferase (ALT), and creatinine in these patients were significantly higher than those in the control group, with this difference being statistically significant (*p* < 0.05). This finding is consistent with the conclusions of previous studies conducted by Li et al. (3), Chan et al. (5), and Zhou et al. (13). Additionally, this study confirmed that a PCT level of ≥5 is an independent risk factor for positive bloodstream bacterial liver abscess in the elderly. Literature indicates that when the body encounters systemic or central nervous system inflammatory responses, the endotoxins released by pathogens can stimulate thyroid C cells, monocytes, and neuroendocrine cells, leading to a substantial increase in PCT secretion. Moreover, the level of PCT is significantly correlated with the severity, progression, or resolution of the infection (4, 14). This study demonstrates that the elevation of PCT levels is more pronounced in PLA patients within the bloodstream-positive group. For patients with compromised immune function or immunodeficiency, the elevated PCT levels provide a more reliable basis for formulating effective treatment strategies (12, 15). Furthermore, the degree of increase in plasma PCT levels may serve as an indicator for predicting the risk of adverse outcomes in patients, such as mortality or the need for admission to the intensive care unit (ICU) (16, 17).

The study found that, compared to the control group, the incidence of complications such as pulmonary infection, pleural effusion, abdominal effusion, and extrahepatic abscesses was significantly higher in the bloodstream-positive group. Among these, pulmonary infection and pleural effusion were the most prevalent complications, a finding that is consistent with the conclusions of the research conducted by Lee et al. (18) and Tian et al. (19). This phenomenon is primarily attributed to the ability of bacteria to disseminate to other tissues and organs via pathways such as blood vessels, lymphatic vessels, or adjacent tissues. Additionally, the lungs, as a crucial organ for receiving venous blood from the systemic circulation, possess capillaries that filter bacterial aggregates from the bloodstream, which may contribute to the high incidence of pulmonary infections. The results of this study indicate that a diameter of less than 5 centimeters for an abscess is a risk factor for the occurrence of BLA, which is consistent with findings from previous research (20). This may be related to the relatively slow accumulation process of pus in smaller abscesses, which allows sufficient time for bacteria to escape from the pus and subsequently infect surrounding tissues or disseminate to other locations via the bloodstream. In contrast, although larger abscesses can accumulate more pus, the higher internal pressure may hinder the bacteria’s ability to traverse the abscess wall and invade adjacent tissues.

The findings of this study reveal that *K. pneumoniae* constitutes the highest proportion in the microbiological profile of liver abscesses, accounting for 87.935%, followed closely by *E. coli*. This observation is in agreement with previous research (5, 9, 21). Considering that *K. pneumoniae* is a common opportunistic pathogen in both the respiratory tract and the gastrointestinal tract, and that the population studied primarily consists of elderly patients who often present with multiple underlying conditions such as diabetes, malignancies, and malnutrition, which contribute to immune dysfunction, this may be a significant factor underlying the persistently high infection rates of *K. pneumoniae* (10, 11). This study also observed that *K. pneumoniae* exhibited susceptibility to most *β*-lactam antibiotics, carbapenems, and quinolone antimicrobial agents, which aligns with the actual clinical treatment outcomes. In contrast, *E. coli* displayed a certain level of resistance to some β-lactam antibiotics, aminoglycosides, and quinolone antimicrobial agents. In this study, it was observed that ESBL-producing bacteria were predominantly composed of *E. coli*, accounting for 75.76%. In patients with ESBL-positive BLA, the incidence of thrombocytopenia, complications such as pleural effusion and ascites, the rate of positive blood cultures, and qSOFA scores ≥2 were significantly higher compared to those with ESBL-negative BLA. Therefore, in elderly patients with secondary biliary diseases accompanied by pleural and peritoneal effusion, elevated bilirubin levels, and thrombocytopenia, a high index of suspicion should be maintained for infection caused by multidrug-resistant *E. coli*. It is noteworthy that the study conducted by Ruiz et al. reported a susceptibility rate of over 90% for *E. coli* to carbapenem antibiotics such as imipenem (22); in contrast, this study found a susceptibility rate of 100%. Overall, *E. coli* demonstrates a high level of susceptibility to carbapenem antibiotics, with a relatively low resistance rate. Therefore, for critically ill patients with severe infections, this class of drugs can be administered as an initial empirical therapy prior to the identification of the causative pathogen, with subsequent adjustments to the treatment regimen based on the antimicrobial susceptibility profile of the isolated pathogen.

The combined diagnostic index for elderly patients with bacteremia-positive BLA yielded an area under the curve (AUC) value of 0.840, with a 95% confidence interval ranging from 0.780 to 0.873. The sensitivity was recorded at 0.766, while the specificity was 0.722. This performance surpasses that of PCT alone at a threshold of ≥5, which demonstrated an AUC of 0.700, indicating a significant predictive efficacy for elderly patients with bacteremia-positive BLA. For patients exhibiting a body temperature of 39°C or higher, with a bacterial liver abscess diameter of 5 centimeters or less, a PCT level of ≥5 ng/mL, and concurrent underlying biliary disease or hepatitis B virus infection, there is a significantly elevated likelihood of positive blood cultures. Therefore, it is imperative to closely monitor the vital signs of these patients and to enhance both anti-infective therapy and symptomatic supportive treatment. This approach aims to prevent hematogenous dissemination, which could lead to severe complications such as extrahepatic abscesses and even septic shock, thereby posing a significant risk to patient mortality.

In summary, in elderly patients with BLA who exhibit positive blood cultures, there is often a presentation characterized by higher body temperatures and elevated PCT levels, along with relatively smaller abscess sizes. Therefore, it is crucial to maintain a high level of vigilance regarding the potential for secondary bloodstream infections in this patient population, with the primary pathogens predominantly consisting of Gram-negative bacteria such as *K. pneumoniae* and *E. coli*. In terms of treatment, it is imperative to actively select antimicrobial agents effective against Gram-negative bacteria, such as carbapenems or *β*-lactamase inhibitor combination formulations, for empirical anti-infection therapy and symptomatic management. Once the patient’s condition improves, the treatment regimen should be adjusted based on the results of susceptibility testing.

## Data Availability

The raw data supporting the conclusions of this article will be made available by the authors, without undue reservation.
